# *Bordetella pertussis* bacteremia in infants co-infected with cytomegalovirus and respiratory syncytial virus

**DOI:** 10.3389/fmicb.2025.1544935

**Published:** 2025-02-19

**Authors:** Wenqiang Sun, Mengzhao Li, Xueping Zhu

**Affiliations:** Department of Neonatology, Children’s Hospital of Soochow University, Suzhou, China

**Keywords:** *Bordetella pertussis*, bacteremia, infant, metagenomic next-generation sequencing, case report

## Abstract

**Introduction:**

Hematogenous infections caused by *Bordetella pertussis* are rare. This study aimed to increase clinicians’ knowledge of *B. pertussis* bacteremia.

**Methods:**

We described a case of an infant with *B. pertussis* bacteremia, searched and reviewed for *B. pertussis* bacteremia-related literatures published in the PubMed database between 1946 to 2022.

**Results:**

A 3-month-old male infant was admitted to the hospital with a respiratory tract infection. Respiratory pathogen testing indicated the presence of *B. pertussis*, cytomegalovirus, and respiratory syncytial viruses. Blood metagenomic next-generation sequencing (mNGS) confirmed *B. pertussis* bacteremia. After 32 days of anti-infective treatment and supportive therapy, the patient’s condition improved, and he was discharged. The literature review found that *B. pertussis* bacteremia is rare, often with fever as the first symptom, and is most common in individuals with underlying diseases or prolonged immunosuppressive therapy.

**Discussion:**

In infants lacking specific protective antibodies against *B. pertussis*, *B. pertussis* bacteremia should be considered when bacteremia-associated clinical manifestations are present and the causative organism remains undetected. Timely refinement of mNGS can help clarify the diagnosis.

## 1 Introduction

*Bordetella pertussis* is a Gram-negative aerobic bacterium transmitted through respiratory droplets and aerosols. It is characterized by acid resistance and slow growth, primarily infecting humans. Carriers of *B. pertussis*, including those with subclinical or latent infections, are the primary sources of transmission (Fry et al., 2021). Over the past decade, the incidence of pertussis has increased significantly in certain regions, with reports from China showing 2.15 cases per 100,000 individuals. Additionally, the global “pertussis resurgence” is being confronted (Chinese Preventive Medicine Association, Vaccine and Immunization Branch of Chinese Preventive Medicine Association, 2021; Zhang et al., 2023). Pertussis is a primary cause of infant illness and mortality worldwide. Upon infecting the ciliated epithelial cells in the respiratory tract, *B. pertussis* produces various virulence factors, including pertussis toxins, tracheal cytotoxins, adenylate cyclase toxins, and adhesins. These virulence factors disrupt ciliary movement and overstimulate the mucosal mechanoreceptors, causing spasmodic coughing and eliciting a sustained immune response in the host (Fry et al., 2021; Lauria and Zabbo, 2023). *B. pertussis* is primarily associated with airway involvement, whereas bloodstream invasion is rare yet potentially dangerous. This clinical rarity may lead to under-recognition of the disease. Therefore, this study aimed to raise clinicians’ awareness of pertussis by presenting a case of infant *B. pertussis* bacteremia and reviewing the relevant literature.

## 2 Materials and methods

### 2.1 Study population

This study was retrospective, and the participants were a male infant who presented with *B. pertussis* bacteremia.

### 2.2 Metagenomic next-generation sequencing

Plasma was prepared from blood samples, and circulating cell-free DNA was isolated from the plasma using the QIAamp Circulating Nucleic Acid Kit (Qiagen, Germany) following the manufacturer’s protocols. The quantity and quality of DNA were assessed using Qubit (Thermo Fisher Scientific, United States) and NanoDrop (Thermo Fisher Scientific, United States), respectively. DNA libraries were prepared using the Hieff NGS OnePot II DNA Library Prep Kit for MGI (Yeasen Biotechnology, China), according to the manufacturer’s protocol. Agilent 2100 was used for quality control, and DNA libraries were sequenced as 50 bp single-end reads on an MGISEQ-200 (BGI, China). No template controls were included from extraction to sequencing. Raw sequencing data were split using bcl2fastq2 (version 2.20), and high-quality data were generated using Trimmomatic (version 0.36) by removing low-quality reads, adapter-contaminated reads, duplicates, and short reads (<36 bp). Human host sequences were eliminated by mapping to the human reference genome (hs37d5) using bowtie2 (version 2.2.6). Reads that could not be mapped to the human genome were retained and aligned with the microorganism genome database for microbial identification using Kraken (version 2.0.7) and for species abundance estimation using Bracken (version 2.5.0). The microorganism genome database contained genomes or scaffolds of bacteria, fungi, viruses, and parasites (downloaded from GenBank release 238, ftp://ftp.ncbi.nlm.nih.gov/genomes/genbank/). The criteria for detection positivity were as follows: (1) at least one species-specific read for the detection of *Mycobacterium*, *Nocardia*, and *Legionella pneumophila*; (2) at least three unique reads required for other bacteria, fungi, viruses, and parasites; and (3) pathogens were excluded if the ratio of microorganism reads per million of a given sample to no template control was <10.

### 2.3 Pathogen Nucleic Acid Detection Kit

Multiplex test kit for 13 respiratory pathogens (fluorescent PCR-capillary electrophoresis method) (Ningbo Hailshi Gene Science and Technology Co., Ltd., Ningbo, China), including influenza A virus, influenza A virus H1N1, influenza A virus H3N2, influenza B virus, parainfluenza virus, rhinovirus, metapneumovirus, coronavirus (excluding corona-virus), respiratory syncytial virus, bocavirus, adenovirus, and *Mycoplasma pneumoniae*, chlamydia pneumoniae. *B. pertussis* Nucleic Acid Test Kit (PCR-Fluorescent Probe Method) (Shenzhen Yicubic Biotechnology Co., Shenzhen, China). Human Cytomegalovirus Nucleic Acid Test Kit (PCR-Fluorescent Probe Method) (Hunan Shengxiang Biotechnology Co., Ltd., Changsha, China). All samples were sent to the laboratory for testing within 2 h after sampling, and specimen handling and testing procedures were performed.

### 2.4 Literature research

A literature search was performed using the PubMed. The search employed the keywords “*Bordetella pertussis*” AND “blood” to find relevant articles.

## 3 Results

### 3.1 Case report

A 3-month-old male was admitted to our Pediatric Intensive Care Unit (PICU) on January 9th, 2021. He had been coughing for 5 days, accompanied by intermittent fever for 2 days and difficulty breathing starting 1 day prior. No abnormalities were noted in his birth, growth, development, family, or medical history. The patient had received only the hepatitis B and Bacillus Calmette–Guérin vaccines after birth and had not yet received any other vaccines. The patient had experienced paroxysmal, spasms-like coughing 5 days prior and had been administered oral antitussive medication; however, the cough did not improve. He had developed a fever (38.2°C) and had sought medical attention at the local community hospital 2 days earlier. A complete blood count showed a white blood cell count of 38.54 × 10^9^/L, with lymphocytes accounting for 0.487, and a platelet count of 718 × 10^9^/L. Bilateral peripheral blood cultures did not reveal bacterial growth after 5 days. After 2 days of treatment with mask oxygen, antibiotics [piperacillin sodium (80 mg/kg, Q8H) and tazobactam sodium (10 mg/kg, Q8H) for injection], peramivir, and methylprednisolone sodium succinate, the child’s clinical symptoms worsened. The treatment was switched to meropenem (10 mg/kg, Q8H) for anti-infection, milrinone was introduced, and immunoglobulin therapy was administered for 1 day. The patient remained irritable and dyspnoeic, had an increased heart rate, and exhibited low blood oxygen saturation during mask oxygenation. He was transferred to the PICU for further treatment.

**Figure 1:**
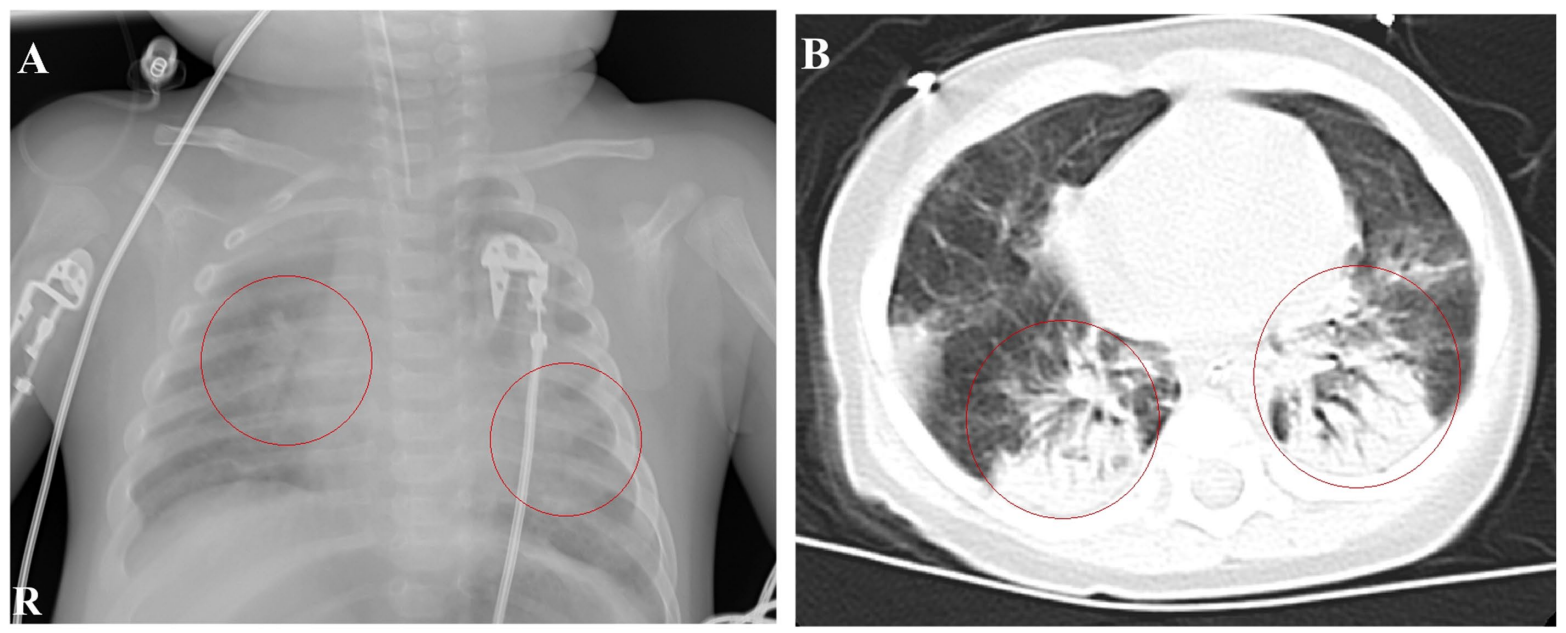
Chest imaging at admission. **(A)** Chest radiograph on January 9th shows patchy shadows in both lungs. **(B)** Chest CT on January 10th reveals multiple patchy hyperdense and solid shadows.

Upon admission, the patient had a body temperature of 38.7°C, heart rate of 200 beats per min, respiratory rate of 60 breaths per min, and blood pressure of 80/56 mmHg (1 mmHg = 0.133 kPa). He exhibited restlessness, cyanosis of the lips, nodding respiration, and inspiratory retraction. Bilateral lung auscultation revealed moist rales, a grade II systolic murmur in the precordial area, and a capillary refill time of <2 s. With mask oxygen inhalation, his SpO_2_ was 92%, as determined by pulse-oximetry. Chest radiography revealed multiple patchy shadows in both lungs (Figure 1A). Chest computed tomography showed multiple patchy hyperdense shadows in both lungs with air bronchogram (Figure 1B). Echocardiography identified an atrial septal defect measuring 2.5 mm. Cranial MRI showed no abnormalities. Routine blood examination revealed a white blood cell count of 60.41 × 10^9^/L, lymphocyte count of 28.45 × 10^9^/L, lymphocyte percentage of 0.471, neutrophil percentage of 0.425, hemoglobin level of 92 g/L, and platelet count of 677 × 10^9^/L. High-sensitivity C-reactive protein (CRP) was measured at 63.99 mg/L. The procalcitonin level was 1.52 ng/mL. Bilateral peripheral blood cultures did not reveal bacterial growth after 5 days. Nucleic acid tests for *B. pertussis* and respiratory syncytial virus (RSV) in the sputum were positive, whereas tests for other respiratory pathogens were negative. Sputum culture did not reveal pathogenic bacteria growth after 5 days. Blood cytomegalovirus (CMV) DNA showed a concentration of 1.56 × 10^4^ copies/mL, and urine CMV DNA was 7.71 × 10^2^ copies/mL. Blood gas analysis revealed an oxygen tension of 58 mmHg, carbon dioxide tension of 50 mmHg, and sodium level of 132 mmol/L. Screening for renal function, humoral and cellular immunity, and genetic metabolic diseases showed no abnormalities. The patient received ventilator-assisted ventilation in pressure-control mode with a positive end-expiratory pressure of 4 cmH_2_O. The infant had a definite *B. pertussis* respiratory infection and was orally administered azithromycin (10 mg/kg/day, orally for 3 days and then stopped for 4 days for three courses). Meropenem (20 mg/kg, Q8H) and ambroxol were administered intravenously, and budesonide, ipratropium bromide, and acetylcysteine via nebulization.

**Figure 2:**
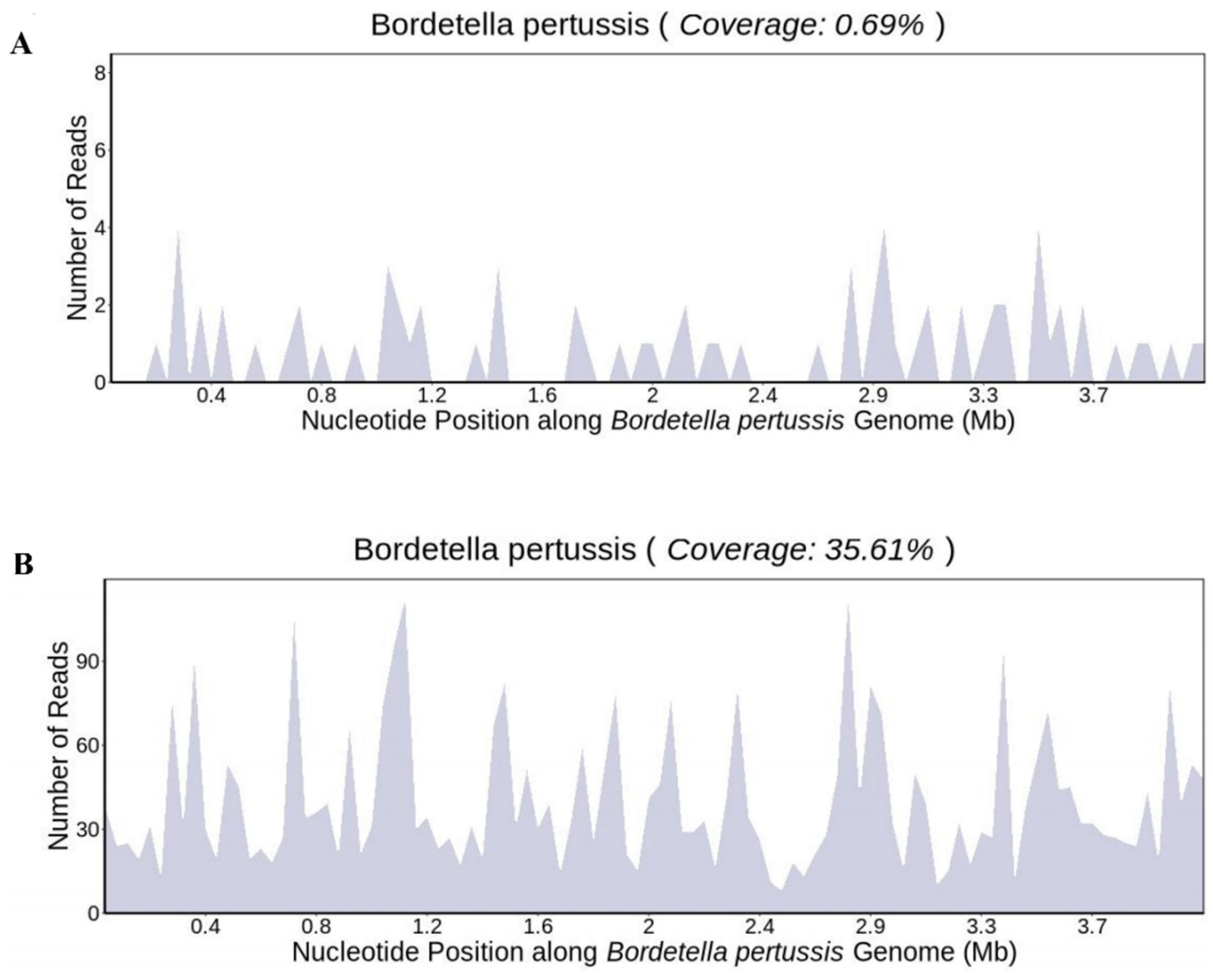
Genome coverage map of *Bordetella pertussis* in **(A)** blood sample and **(B)** bronchoalveolar lavage fluid.

After 1 day of treatment in the PICU, the patient developed a persistent high fever. The highest recorded body temperature was 39.7°C, accompanied by recurrent vomiting and a deterioration in mental responsiveness. The capillary refill time was measured at 3 s. Routine cerebrospinal fluid and biochemical analyses revealed no abnormalities. The cerebrospinal fluid showed no bacterial growth in culture for up to 5 days. On January 10th, peripheral blood and bronchoalveolar lavage fluid metagenomic next-generation sequencing (mNGS) tests were completed, and linezolid was added to address the infection because the infection was not effectively controlled. On January 12th, 24 unique sequence reads of *B. pertussis* were detected in peripheral blood mNGS, accounting for 0.69% of genome coverage (Figure 2A). mNGS performed on bronchoalveolar lavage fluid showed 1,956 unique sequence reads of *B. pertussis*, accounting for 35.61% of genome coverage (Figure 2B). Additionally, CMV was detected in the blood and bronchoalveolar lavage fluid. The species detected by mNGS are listed in Table 1. The child’s clinical presentation, routine blood tests, and inflammatory indices indicated a likelihood of bacteremia. The diagnosis of *B. pertussis* bacteremia was considered using mNGS sequencing results.

**Table 1:** mNGS results of peripheral blood and bronchoalveolar lavage fluid.

Sample	Kingdom	Genus	Species	Species unique reads	Coverage
Blood	Bacterium	*Bordetella*	*Bordetella pertussis*	24	0.69%
	Virus	*Cytomegalovirus*	*Human betaherpesvirus 5*	49	1.25%
Bronchoalveolar lavage fluid	Bacterium	*Bordetella*	*Bordetella pertussis*	1,956	35.61%
	Virus	*Cytomegalovirus*	*Human betaherpesvirus 5*	20	0.53%
	Fungi	*Pneumocystis*	*Pneumocystis jirovecii*	271	0.18%
	Fungi	*Candida*	*Candida parapsilosis*	3	0.0029%

mNGS, metagenomic next-generation sequencing.

**Figure 3:**
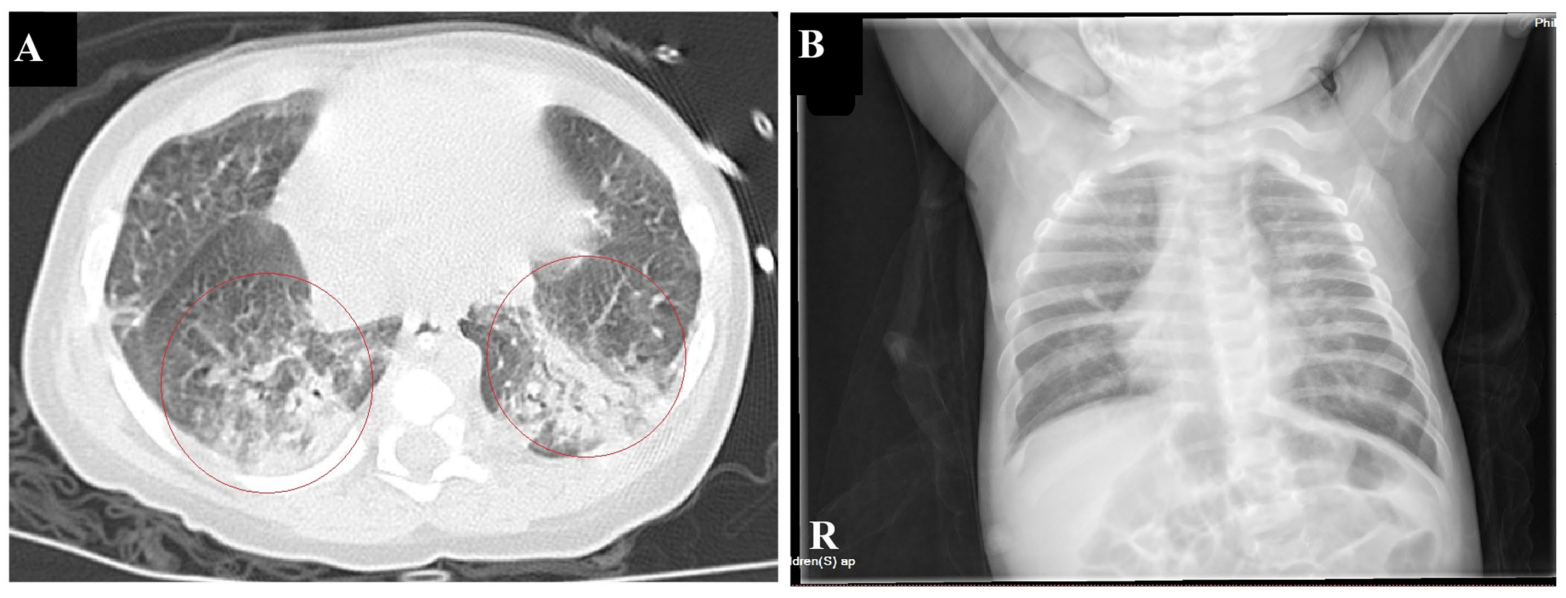
Chest imaging before discharge **(A)**. Chest CT on January 23rd shows a significant reduction in patchy hyperdense shadows in both lungs compared to the previous imaging **(B)**. Chest radiographs on February 9th indicate a significant improvement in lung inflammation.

The infant had coinfections with *B. pertussis*, RSV, and CMV, with *B. pertussis* invading the bloodstream. The infant’s vital signs are stable and the degree of infection has improved significantly; therefore, linezolid was discontinued, and azithromycin and meropenem were continued. A repeat blood test indicated a hemoglobin level of 63 g/L, and 1.0 U of red blood cell suspension leukocytes-reduced was administered via infusion. On January 16th, the patient showed notable clinical improvement. Routine blood examination revealed a white blood cell count of 19.54 × 10^9^/L and CRP levels of 10.85 mg/L. Meropenem was discontinued, and the patient was switched to cefoperazone sulbactam (50 mg/kg, Q8H). By January 21st, the patient’s breathing and heart rate had stabilized. A repeat blood test showed a white blood cell count of 15.82 × 10^9^/L (with lymphocytes accounting for 0.571), platelet count of 431 × 10^9^/L, hemoglobin of 73 g/L, and CRP of 3.68 ng/mL. Additionally, 1.0 U of red blood cell suspension leukocytes-reduced was given by infusion. The patient was extubated and switched to high-flow oxygen therapy. On January 23rd, sputum and wet rales in both lungs significantly improved. A repeat blood test showed a white blood cell count of 11.06 × 10^9^/L, with lymphocytes accounting for 0.674, platelet count of 435 × 10^9^/L, and CRP at 2.99 ng/mL. Chest computed tomography indicated significant improvement in inflammation in both lungs (Figure 3A). Cefoperazone-sulbactam was discontinued and replaced with cefozoxime to prevent infection.

**Figure 4:**
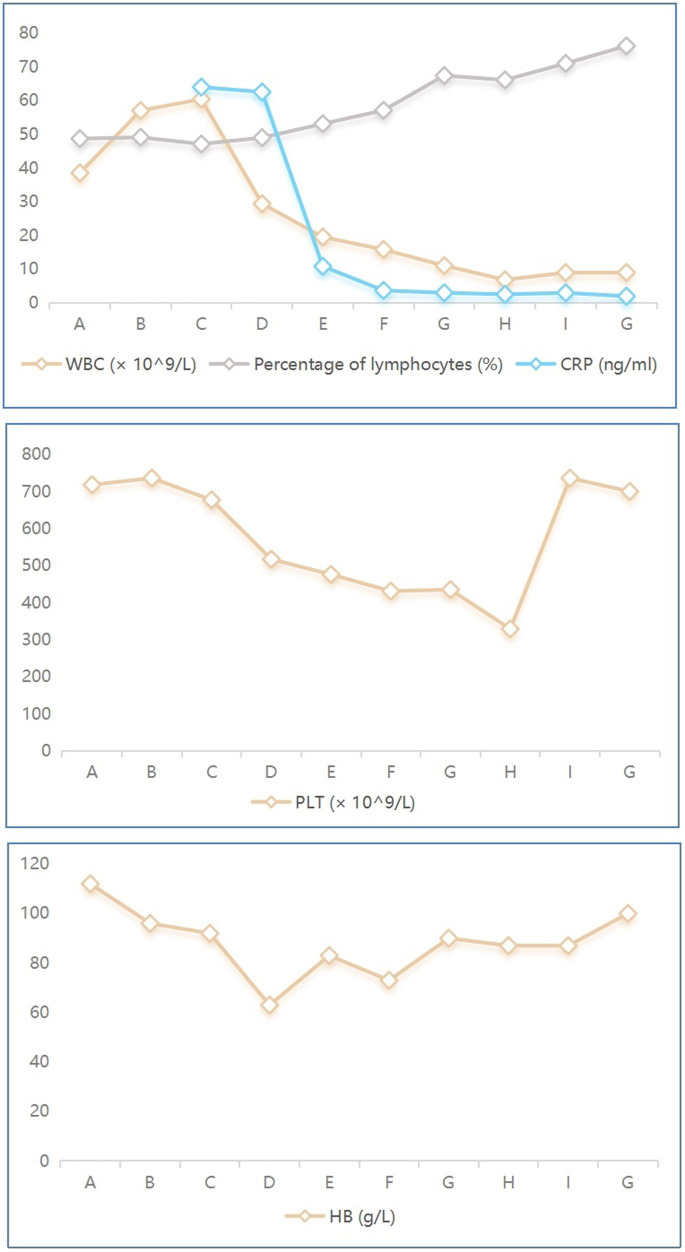
Dynamic laboratory indicators in the infant with *Bordetella pertussis*. A–G denotes the date of laboratory testing, 07/01/2021, 09/01/2021, 10/01/2021, 12/01/2021, 16/01/2021, 20/01/2021, 23/01/2021, 26/01/2021, 30/01/2021, 09/02/2021, respectively. WBC, white blood cell; CRP, C-reactive protein; PLT, platelet; HB, hemoglobin.

**Figure 5:**
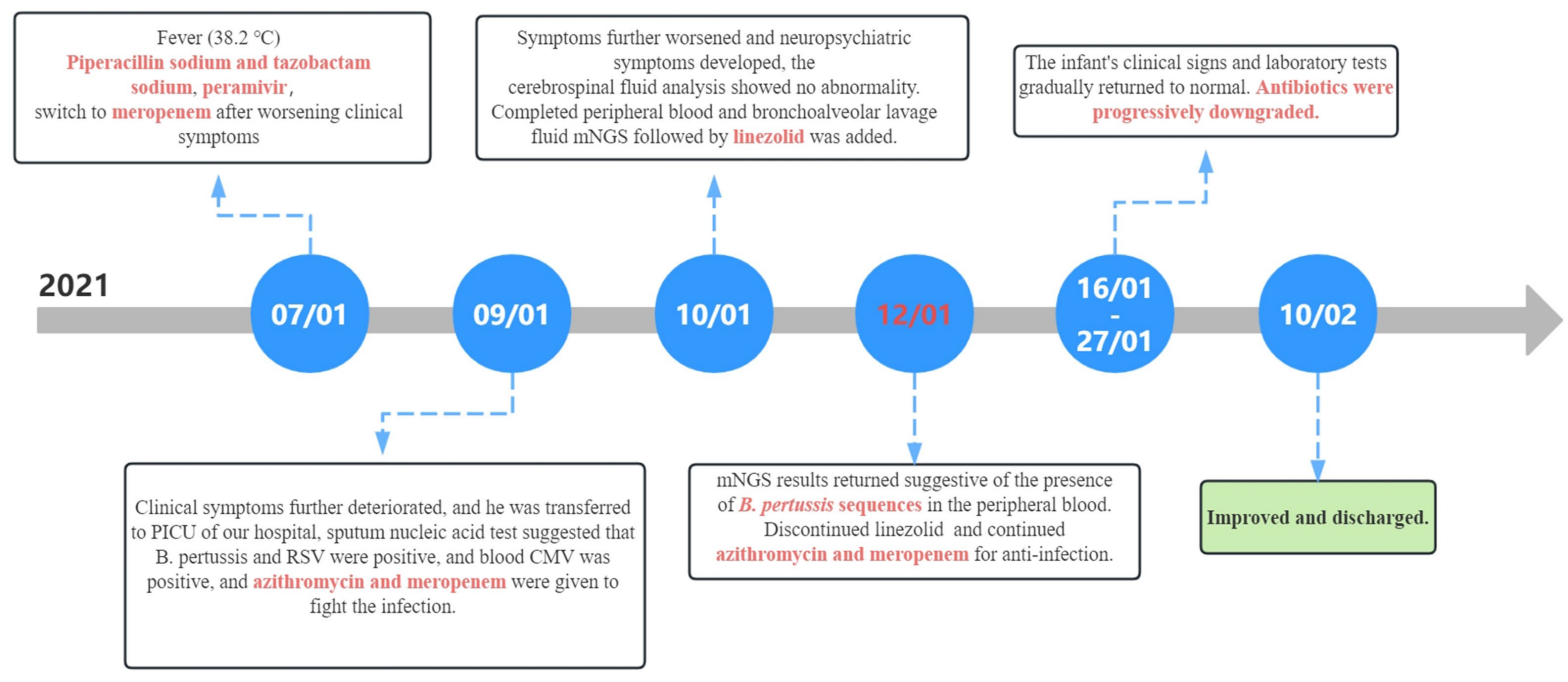
Timeline of disease progression and treatment. CMV, cytomegalovirus; mNGS, metagenomic next-generation sequencing.

On January 27th, the infant’s general condition was good, with only a few sputum sounds heard in both lungs. The patient was switched to nasal catheter oxygen inhalation therapy. On January 30th, oxygen therapy was discontinued. Repeat blood tests showed a white blood cell count of 8.97 × 10^9^/L, lymphocytes accounting for 0.710, hemoglobin of 90 g/L, and CRP of 2.98 ng/mL, with the current treatment regimen continued. On February 10th, the patient experienced notable relief from clinical symptoms. Laboratory indicators returned to normal, and chest imaging showed substantial resolution of lung inflammation compared to previous examinations (Figure 3B). Based on these positive findings, the patient was discharged from hospital. The child’s guardian was satisfied with the doctor’s attitude and the treatment process. The dynamic results of the laboratory tests of the infants are presented in Table 2 and Figure 4. The critical treatment schedule for infants is shown in Figure 5.

**Table 2:** Laboratory tests for the infant of *Bordetella pertussis* bacteremia.

Dates	07/01	09/01	10/01	12/01	16/01	20/01	23/01	26/01	30/01	09/02
WBC (×10^9^/L)	38.54	57.00	60.41	29.40	19.54	15.82	11.06	6.84	8.97	9.01
NE percentage (%)	39.10	38.81	42.50	43.00	32.60	30.30	23.40	22.90	21.80	14.70
LY percentage (%)	48.70	49.10	47.10	49.00	53.10	57.10	67.40	66.10	71.00	76.20
PLT (×10^9^/L)	718	736	677	517	476	431	435	329	736	700
HB (mg/L)	112	96	92	63	83	73	90	87	87	100
CRP (ng/mL)	—	—	63.99	62.53	10.85	3.68	2.99	2.54	2.98	1.98

WBC, white blood cell; NE, neutrophil; LY, lymphocyte; PLT, platelet; HB, hemoglobin; CRP, C-reactive protein.

### 3.2 Literature review results

A total of 3,028 articles were retrieved from the literature; 3,022 were excluded, and 6 articles met the criteria. To date, six cases of *B. pertussis* bacteremia have been reported in the literature (from 1946 to 2022). In this report, these seven cases have been summarized in Table 3 (Janda et al., 1994; Centers for Disease Control and Prevention, 2004; Troseid et al., 2006; Liotti et al., 2021; Wakimoto et al., 2022; Liao et al., 2022).

**Table 3:** Clinical characteristic of six patients with *Bordetella pertussis* bacteremia.

References	Patient 1	Patient 2	Patient 3		Patient 4	Patient 5	Patient 6	Patient 7
	Janda et al. (1994)	Centers for Disease Control and Prevention (2004)	Troseid et al. (2006)		Liotti et al. (2021)	Wakimoto et al. (2022)	Liao et al. (2022)	Present case
Age	31	82	63		3-month-old	49	2-month-old	3-month-old
Sex	Male	Female	Male		Male	Male	Male	Male
Underlying disease	Granulomatosis	MM	MM		Post-ischemic epileptic encephalopathy, Thrombophilia, Silver tube tracheostomy	HIV	Low birth weight, Very preterm	Normal
Symptom	Dry cough, fever, progressive shortness of breath, wheezing	Fever (39.0°C), cough, dyspnea	1st admission: fever (39.7°C), productive cough	2nd admission: fever (38.8°C), dry cough, hoarseness	Fever (38.5°C), tachypnic	Fever (38.8°C), productive cough, dyspnea, chest pain	Fever (37.8°C), mild cough, tachypnea, triple concave signs, mild cyanosis of the lips	Dry cough, fever, progressive shortness of breath, wheezing
Lung conditions	Pneumonia	Pneumonia	Bronchiolitis	Diffuse bilateral wheezing	NA	Pneumonia	Pneumonia	Pneumonia
WBC count	23,200/μL	NA	Normal	12,700/μL	21,760/μL	69,910/μL	Normal	23,200/μL
Chest radiography	Right lower lung infiltrates, a cavity bleb in the right middle lobe	Diffuse left lung infiltrates	Normal		NA	Left upper lung infiltrates, followed by fibrous organization left lower lung nodular shadow	Indistinct reticular shadows in both lungs	Multiple patchy shadows in both lungs
Other pathogens	NA	NA	*Moraxella catarrhalis*	NA	NA	HIV, *Haemophilus influenzae*, small gram-negative bacilli	Negative	NA
Infection therapy	Ceftizoxime, erythromycin, gentamicin	Multiple nonmacrolide drugs	Cefotaxime	Cefotaxime, Benzylpenicillin, clarithromycin	Azithromycin	Ceftriaxone, levofloxacin, azithromycin	Piperacillin tazobactam, meropenem, vancomycin, levofloxacin	Ceftizoxime, erythromycin, gentamicin
Outcome	Died	Died	Cured		Cured	Cured	Cured	Cured

MM, multiple myeloma; HIV, human immunodeficiency virus; WBC, white blood cell; NA, not available; CMV, cytomegalovirus; RSV, respiratory syncytial virus.

## 4 Discussion

*B. pertussis* typically invades and persists within the ciliated epithelial cells of the airway. Hematogenous infections are rare and have been reported primarily in adults with neoplasia, acquired immunodeficiency, or long-term use of immunosuppressive drugs. These patients commonly present with fever or intermittent febrile episodes (Janda et al., 1994; Centers for Disease Control and Prevention, 2004; Troseid et al., 2006; Wakimoto et al., 2022). Additionally, the literature includes reports of *B. pertussis* infection leading to encephalitis, such as in a 6-year-old girl (Di Camillo et al., 2021). Only two cases of caused *B. pertussis* bloodstream infection in infants have been reported to date. However, one case involved an extremely premature infant (born at 31 + 5 weeks) with a birth weight of 1,700 g (Liao et al., 2022). The infection was more severe than in our case, and the possibility of immunodeficiency or an inherited metabolic disease could not be definitively ruled out based on the case report. Another case involved an infant with post-ischemic epileptic encephalopathy and thrombophilia, long-term oral antiepileptic medication, and respiration through a silver tube tracheostomy (Liotti et al., 2021). Detection of *B. pertussis* in blood using mNGS is rare. It is highly unlikely that *B. pertussis* detected by mNGS belongs to a background source; relatively high sequence detection by mNGS suggests the presence of true nucleic acid sequences of *B. pertussis* in the sample (Gu et al., 2019). In our case, we first detected *B. pertussis* in the patient’s sputum using polymerase chain reaction (PCR) and subsequently in the lung lavage fluid and blood using mNGS. Based on the clinical manifestations and laboratory findings, the diagnosis was *B. pertussis* bacteremia.

In this case, the child initially presented with a paroxysmal spasmodic cough. The disease progressed rapidly with pronounced pulmonary signs. Chest imaging indicated severe pneumonia, while sputum pathogenic nucleic acid testing and alveolar lavage fluid mNGS suggested *B. pertussis* infection. During pneumonia treatment, the child gradually developed a high fever, impaired mental responsiveness, and prolonged capillary refill time. In summary, we speculate that this hematogenous infection originated from the invasion of *B. pertussis* from the lungs into the bloodstream. However, the mechanism through which *B. pertussis* invades the bloodstream from the lungs remains unclear. Previously reported adult cases had immunodeficiencies and underlying diseases. In this case, immunodeficiency was not considered a factor, as the child exhibited normal growth and development, no history of recurrent infections or eczema, no abnormalities in humoral and cellular immunity, genetic metabolism screening after admission, and no family history of immunodeficiency. However, the child was young and had not received vaccination against *B. pertussis*. Additionally, the mother did not receive protective vaccines during pregnancy, resulting in a lack of specific protective antibodies. The child was coinfected with RSV and CMV. Lung damage mediated by RSV and CMV may have contributed to the invasion of *B. pertussis* into the bloodstream. Studies have demonstrated that CMV directly contributes to lung injury and diminishes overall and local immunity in the lungs. These effects can potentially compromise mucosal barrier function and provide favorable conditions for the invasion of *B. pertussis* into the bloodstream (Vergara et al., 2018). RSV is a prevalent pathogen in pertussis combined with viral infections. Concurrent RSV infections substantially exacerbate the severity of pertussis, leading to more pronounced pulmonary symptoms and increased rates of hospitalization and rehospitalization (Zhang and Deng, 2021). These findings suggest that RSV may contribute not only to severe pertussis but also to the invasion of *B. pertussis* into the bloodstream. Therefore, our final assessment suggests that the absence of specific protective antibodies, lung injury mediated by RSV and CMV, and elevated levels of *B. pertussis* bacterial load in the lungs collectively contribute to the progression of *B. pertussis* invasion into the bloodstream.

Whooping cough-like symptoms are more commonly observed in infants than in adults. In the early stages of the disease, paroxysmal spasmodic cough may appear, enabling the timely detection of *B. pertussis* infections. However, *B. pertussis* invasion into the bloodstream is infrequent and often overlooked in clinical practice. *B. pertussis* has a slow growth rate and typically requires a minimum of 6 days for detection through routine peripheral blood cultures. However, laboratory blood cultures are commonly conducted within a standard time frame of 5 days, which can potentially result in missed diagnoses (Wakimoto et al., 2022). Additionally, the infant was treated with antibiotics before peripheral blood was collected for blood culture, which may have been a primary reason for the failure to detect *B. pertussis* in the blood culture. In patients with immunodeficiency or chronic immunosuppression, the possibility of *B. pertussis* bacteremia should be considered even in cases where blood cultures yield negative results. In infants and young children with *B. pertussis* pneumonia, particular attention should be paid to sepsis-related manifestations, especially in the presence of concurrent viral infections such as RSV or CMV. In such cases, it is recommended to extend the duration of the blood culture and, if necessary, employ real-time PCR or mNGS technology as early as possible.

PCR technology enables the prompt, sensitive, and specific detection of exogenous genes that invade the body; however, it is highly sensitive, and even minimal contamination of target genes can lead to inaccurate results, resulting in a high rate of false positives. Moreover, PCR technology does not definitively determine whether a pathogen is viable, nor does it indicate whether the nucleic acids of the detected pathogen are expressed (Wilson et al., 2014). In this study, we detected *B. pertussis* in sputum using PCR, which provided a relevant basis for further examination. Unfortunately, we initially did not consider the possibility of *B. pertussis* invading the bloodstream and, therefore, did not use PCR for peripheral blood *B. pertussis* determination. mNGS is sensitive, time-efficient, and offers advantages in diagnosing “unknown” pathogens and infections involving multiple mixed pathogens. Additionally, mNGS is less affected by antibiotics than conventional culture methods, reducing the likelihood of false-negative pathogen detection owing to the early empiric use of antibiotics. The results of mNGS can further guide the use of antibiotics. In this study, after empirical antibiotic administration, children underwent peripheral blood and bronchoalveolar lavage fluid mNGS, which detected relevant pathogenic bacteria after 2 days. Subsequently, we adjusted the antibiotic regimen and achieved better therapeutic results, highlighting the advantages of mNGS in detecting pathogenic microorganisms in infectious diseases. A key disadvantage of mNGS is that the microbial nucleic acids in most patient samples have a predominantly human host background. The vast majority of reads (typically >99%) are from human hosts, thus limiting the overall analytical sensitivity of pathogen detection methods because of the relative scarcity of sequenced microbial non-human reads. This inherent limitation of unbiased mNGS can be partially mitigated by using targeted sequencing or host-depletion methods (Gu et al., 2016; Hasan et al., 2016). mNGS technology encounters issues related to contamination control, low nucleic acid extraction efficiency, non-standardized sequencing, bioinformatic analysis procedures, and varying levels of report interpretation (Gu et al., 2019; Edward and Handel, 2021). In clinical practice, a comprehensive analysis of mNGS results, alongside epidemiological and clinical characteristics, is essential. Integrating multiple testing methods is necessary to identify the true causative organisms.

This study has some limitations. It was a single case report and literature review, and despite our efforts to include as much relevant literature as possible, the number of cases remains small. Additionally, we did not perform bacterial cultures prior to antibiotic administration and cultured the bacteria for only 5 days, which may explain why we were unable to culture *B. pertussis*.

## 5 Conclusion

We described a case of *B. pertussis* bacteremia in an infant co-infected with RSV and CMV. *B. pertussis* bacteremia is uncommon and mainly occurs in individuals with neoplasia, immunodeficiency, or prolonged immunosuppressive therapy. This case highlights the importance of considering *B. pertussis* bacteremia in infants and children lacking specific protective antibodies against *B. pertussis*, particularly when they present with bacteremia in the context of mixed viral infections. Early use of mNGS can facilitate prompt diagnosis and precise treatment, thus avoiding excessive testing and therapeutic interventions.

## Data Availability

The original contributions presented in the study are included in the article/supplementary material, further inquiries can be directed to the corresponding author.

## References

[ref1] Centers for Disease Control and Prevention (2004). Fatal case of unsuspected pertussis diagnosed from a blood culture—Minnesota, 2003. MMWR Morb. Mortal Wkly. Rep. 53, 131–132. 14981363

[ref2] Chinese Preventive Medicine Association, Vaccine and Immunization Branch of Chinese Preventive Medicine Association (2021). Expert consensus on China’s pertussis action plan. Chin. J. Epidemiol. 42, 955–965. doi: 10.3760/cma.j.cn112338-20210308-00186 (in Chinese)

[ref3] Di Camillo C. Vittucci A. C. Antilici L. Ciarlitto C. Linardos G. Concato C.. (2021). Pertussis in early life: underdiagnosed, severe, and risky disease. A seven-year experience in a pediatric tertiary-care hospital. Hum. Vaccin. Immunother. 17, 705–713. doi: 10.1080/21645515.2020.1791617 32755440 PMC7993225

[ref4] Edward P. Handel A. S. (2021). Metagenomic next-generation sequencing for infectious disease diagnosis: a review of the literature with a focus on pediatrics. J. Pediatric Infect. Dis. Soc. 10, S71–S77. doi: 10.1093/jpids/piab104, PMID: 34951466

[ref5] Fry N. K. Campbell H. Amirthalingam G. (2021). JMM Profile: *Bordetella pertussis* and whooping cough (pertussis): still a significant cause of infant morbidity and mortality, but vaccine-preventable. J. Med. Microbiol. 70:70. doi: 10.1099/jmm.0.001442 PMC8604168 34668853

[ref6] Gu W. Crawford E. D. O'Donovan B. D. Wilson M. R. Chow E. D. Retallack H.. (2016). Depletion of Abundant Sequences by Hybridization (DASH): using Cas9 to remove unwanted high-abundance species in sequencing libraries and molecular counting applications. Genome Biol. 17:41. doi: 10.1186/s13059-016-0904-5 26944702 PMC4778327

[ref7] Gu W. Miller S. Chiu C. Y. (2019). Clinical metagenomic next-generation sequencing for pathogen detection. Annu. Rev. Pathol. 14, 319–338. doi: 10.1146/annurev-pathmechdis-012418-012751 30355154 PMC6345613

[ref8] Hasan M. R. Rawat A. Tang P. Jithesh P. V. Thomas E. Tan R.. (2016). Depletion of human DNA in spiked clinical specimens for improvement of sensitivity of pathogen detection by next-generation sequencing. J. Clin. Microbiol. 54, 919–927. doi: 10.1128/JCM.03050-15, PMID: 26763966 PMC4809942

[ref9] Janda W. M. Santos E. Stevens J. Celig D. Terrile L. Schreckenberger P. C. (1994). Unexpected isolation of *Bordetella pertussis* from a blood culture. J. Clin. Microbiol. 32, 2851–2853. doi: 10.1128/jcm.32.11.2851-2853.1994, PMID: 7852585 PMC264173

[ref10] Lauria A. M. Zabbo C. P. (2023). “Pertussis” in StatPearls (Treasure Island, FL: StatPearls Publishing).

[ref11] Liao Y. Li W. R. Zhu Y. Luo S. H. Liao Q. Wan C. M. (2022). Invasive *Bordetella pertussis* infection in infants: a case report. Open Forum Infect. Dis. 9:ofac478. doi: 10.1093/ofid/ofac478, PMID: 36225748 PMC9547520

[ref12] Liotti F. M. De Angelis G. Speziale D. Morandotti G. A. Genovese O. Sanguinetti M.. (2021). *Bordetella pertussis* DNA detected in a tracheostomized child blood before seroconversion. Eur. J. Clin. Microbiol. Infect. Dis. 40, 205–208. doi: 10.1007/s10096-020-03988-4 32661807

[ref13] Troseid M. Jonassen T. O. Steinbakk M. (2006). Isolation of *Bordetella pertussis* in blood culture from a patient with multiple myeloma. J. Infect. 52, e11–e13. doi: 10.1016/j.jinf.2005.04.014, PMID: 15936087

[ref14] Vergara A. Cilloniz C. Luque N. Garcia-Vidal C. Tejero J. Perello R.. (2018). Detection of human cytomegalovirus in bronchoalveolar lavage of intensive care unit patients. Eur. Respir. J. 51:1701332. doi: 10.1183/13993003.01332-2017, PMID: 29437938

[ref15] Wakimoto Y. Otsuka N. Yanagawa Y. Koide K. Kamachi K. Shibayama K.. (2022). The first reported case of *Bordetella pertussis* bacteremia in a patient with human immunodeficiency virus infection. Open Forum Infect. Dis. 9:ofac20. doi: 10.1093/ofid/ofac020 PMC8825563 35146052

[ref16] Wilson M. R. Naccache S. N. Samayoa E. Biagtan M. Bashir H. Yu G.. (2014). Actionable diagnosis of neuroleptospirosis by next-generation sequencing. N. Engl. J. Med. 370, 2408–2417. doi: 10.1056/NEJMoa1401268, PMID: 24896819 PMC4134948

[ref17] Zhang R. Deng J. (2021). Clinical impact of respiratory syncytial virus infection on children hospitalized for pertussis. BMC Infect. Dis. 21:161. doi: 10.1186/s12879-021-05863-9, PMID: 33563205 PMC7871314

[ref18] Zhang M. Wu D. Li Y. X. Zheng H. Yin Z. D. Liang X. F. (2023). Challenges to global pertussis prevention and control. Zhonghua Liu Xing Bing Xue Za Zhi 44, 491–497. doi: 10.3760/cma.j.cn112338-20220825-00737 36942347

